# Harder than Expected: Increased Conflict in Clearly Disadvantageous Delayed Choices in a Computer Game

**DOI:** 10.1371/journal.pone.0079310

**Published:** 2013-11-15

**Authors:** Stefan Scherbaum, Maja Dshemuchadse, Susanne Leiberg, Thomas Goschke

**Affiliations:** 1 Department of Psychology, Technische Universität Dresden, Dresden, Germany; 2 Department of Economics, University of Zurich, Zurich, Switzerland; University of Sheffield, United Kingdom

## Abstract

When choosing between immediate and temporally delayed goods, people sometimes decide disadvantageously. Here, we aim to provide process-level insight into differences between individually determined advantageous and disadvantageous choices. Participants played a computer game, deciding between two different rewards of varying size and distance by moving an agent towards the chosen reward. We calculated individual models of advantageous choices and characterized the decision process by analyzing mouse movements. The larger amount of participants’ choices was classified as advantageous and the disadvantageous choices were biased towards choosing sooner/smaller rewards. The deflection of mouse movements indicated more conflict in disadvantageous choices compared with advantageous choices when the utilities of the options differed clearly. Further process oriented analysis revealed that disadvantageous choices were biased by a tendency for choice-repetition and an undervaluation of the value information in favour of the delay information, making rather simple choices harder than could be expected from the properties of the decision situation.

## Introduction

Human choices sometimes deviate from rationality standards as defined, for instance, by the economical rule of utility maximization [1]. A prominent example is intertemporal choice, where individuals choose between smaller rewards delivered sooner and larger rewards delivered later. Empirical studies found that, compared to normative theories [2], individuals often discount rewards too steeply, especially for the near future [3]–[6]. These findings raised the question whether the economic norm, originally stated as a first assumption [2], failed or whether individuals choose disadvantageously. In the following, a wide range of research aimed to close this explanatory gap from two sides: First by searching for discounting models with a better data-fit [7], for example by adding psychological assumptions like a logarithmic instead of a linear scaling of time [8], [9]; and second by explaining why individuals choose disadvantageously, for example by assuming impulsive and less reflective choices [10], [11]. However, the prior question whether intertemporal choice behavior is advantageous or disadvantageous from a normative view remained unsolved. Moreover, due to the lack of a valid economic reference model in common tasks, little is known about why humans fail to choose advantageously. Do they indeed, as often assumed [12], chose against better knowledge or are they just committing errors?

**Figure 1:**
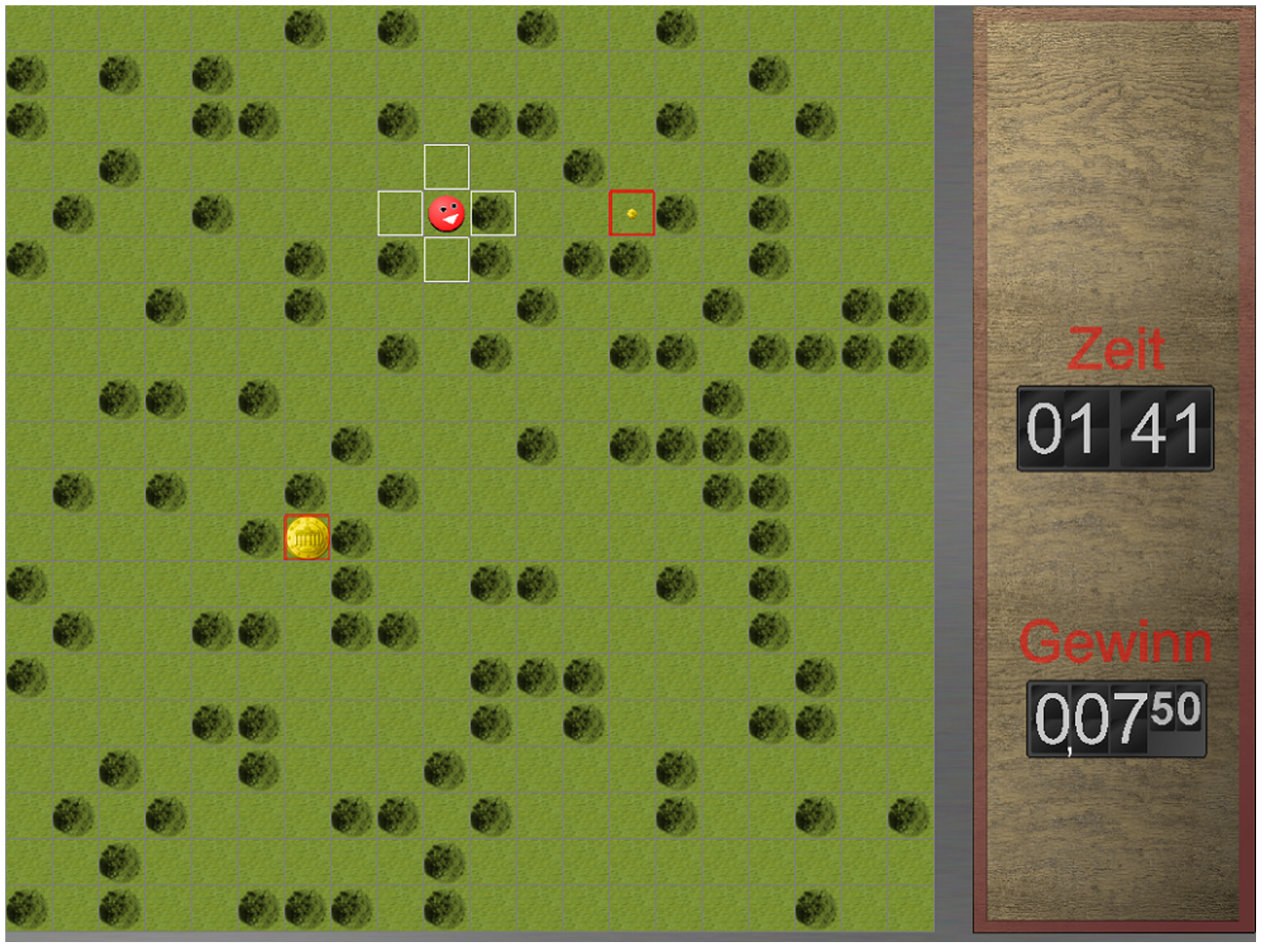
The experimental screen. Participants chose between soon/small and late/large rewards (coins of different size with a red border), moving an agent (red smiling face) across a playing field by clicking with the mouse into horizontally or vertically adjacent fields (white border). They were instructed to maximize their gain within the limited time of 8 minutes per block. The remaining time (“Zeit”) within a block and the cumulated credits (“Gewinn”) were presented next to the playing field.

Out of these considerations, we will present an approach to intertemporal choice based on participant-specific models of advantageous choices and the registration of within-trial choice action dynamics to compare the decision process leading to advantageous and disadvantageous choices. We designed a delay discounting computer game (see Figure 1) in which participants had a restricted time to collect rewards by moving an agent on a playing field via a computer mouse. This paradigm enabled us to compute a decision model of advantageous choices adapted to the individual participants’ speed of decision and movement. According to this model, we were able to distinguish advantageous and disadvantageous choices. Furthermore, to investigate within-trial choice dynamics of advantageous and disadvantageous choices, we recorded mouse movement trajectories [13]. Previous studies have shown that such a continuous approach [14] can reveal conflict experienced when deciding between different options [15] and even changes in mind [16]. Less certain and more conflict-laden decisions are reflected in less direct choice trajectories during the decision process [17]. While we investigated this effect previously for choice trajectories of sooner/smaller choices and later/larger choices [18], our novel intertemporal choice task and the participant-specific advantageous decision model enabled us to compare choice trajectories between disadvantageous and advantageous choices. Thus, we were able to investigate if disadvantageous options were chosen against better knowledge or if they were chosen by error: if they were chosen against better knowledge, the tendency to choose disadvantageously should be in conflict to the better knowledge of what should be chosen. Furthermore, the clearer the advantageous option is defined, the greater this conflict should become, indicated by increased deflection of choice trajectories [15], [16], [18]. In contrast, if they were chosen simply due to error, erroneous choices should indicate less conflict within the decision process [19], indicated by less deflected choice trajectories. Building on this insight into the decision processes, we further elaborated the picture of the dimension weighing dynamics (e.g. delays and values) in the decision process and of the influences leading to disadvantageous decisions, we applied time-continuous multiple regression analysis to mouse movements.

Taken together, we expected the larger part of decisions to be advantageous. For the remaining disadvantageous choices, we anticipated a bias towards choosing the sooner/smaller option in concordance with previous research [20]. Furthermore, we expected that individuals sometimes choose disadvantageously against better knowledge [12] reflected in more conflict and hence less direct choice movements for disadvantageous choices compared to advantageous choices. We expected this difference to be especially pronounced when the utilities between the options differed clearly and the advantageousness of the one option should be clear-cut hence inducing strong attractiveness acting against the disadvantageous choice tendency. In contrast, for only slightly differing utilities and, hence, difficult choices, we expected moderate conflict for disadvantageous as well as for advantageous choices, due to increased task difficulty [21]. As has been shown previously [18], [22].

## Methods

The study was performed in accordance with the guidelines of the Declaration of Helsinki and of the German Psychological Society. An ethical approval was not required since the study did not involve any risk or discomfort for the participants. All participants were informed about the purpose and the procedure of the study and gave written informed consent prior to the experiment. All data were analyzed anonymously.

In the experiment, 25 participants (15 female, mean age 23.04 years) played a choice computer game on a 17 inch screen running at a resolution of 1280×1024 pixels (controlled by the Psychophysics Toolbox 3 [23], [24] in Matlab 2006, the Mathworks Inc). In this game, participants were instructed to collect as much reward as possible in the given, limited amount of time by navigating an agent on a playing field divided into 20×20 fields. To move the agent, participants clicked with the mouse (Logitech Laser Mouse USB, 92 Hz sampling frequency) in one of the horizontally or vertically adjacent fields. Next to the playing field, participants could see the remaining amount of time within a block (8 minutes) and the credits already collected. As payment, participants received 3 Euro plus the credits earned in the experiment (*M* = 2.45 Euro, *SE* = 0.05 Euro).

Each trial began with an inter trial interval of 1.3 seconds in which the mouse cursor was locked to the field of the avatar. Afterwards, two reward options were presented as coins: One reward being near, but small, the other far, but large. The size of the coin represented the reward value of the option. Reward values ranged from one to ten credits, with the reward values of the two options in each trial adding up to eleven credits to keep the overall value of each trial constant (smaller/larger reward pairs were: 1/10, 2/9, 3/8, 4/7, 5/6). Distances ranged from one to fourteen fields with the nearer option being at a distance of 1, 3, or 7 fields and the additional interval to the farer option being 1, 2, 4, or 7 fields. Reward values, smaller distance and interval to the larger distance were varied orthogonally with a random order of trials. For better understanding, we maintain in the following the standard description of the time dimension using “soon”, “late”, “delay”, and “interval”, although in our game time delay is represented by spatial distance.

To investigate participants’ decision process in more detail by tracing mouse movements, we ensured that the two options were always placed in a way that the first click of a participant into a movement field decreased the distance to one option and increased it to the other option (for details, see Material S1). Hence, with their first click, participants already had to decide which option to go for. When participants finally reached the field of one option, this option was collected and both options disappeared.

The experiment consisted of three blocks, with one block lasting eight minutes, allowing participants to work through several repetitions of all combinations of values and distances. Overall, we generated a pool of 480 trials, consisting of 8 randomized sub-blocks containing every delay-value combination. From pretests, we expected this to be enough so that participants would run into the time limit before completing all possible trials.

## Results

On average, participants completed 351 trials (*SE = *7). Hence, every delay-value combination was dealt with almost 6 times. To evaluate participants’ performance, we determined the advantageous choice for each trial by determining which of the two options yielded the better cost/benefit ratio (for details, see Material S1). While the benefit of each option was defined by the amount of credits for this option, we calculated the hypothetical costs for each option based on the individual time profile of each participant (see Material S1). We first analyzed the time needed to reach the chosen option clicking from field to field. The first (deciding) click took on average 770 ms (*SD* = 25 ms), while all following clicks needed an average time of 359 ms (*SD* = 84 ms). This difference between the first and following clicks, and a very low number of trials showing a change of mind after the first click (*M* = 0.9%, *SD* = 6%) indicated that participants continued to pursue the option chosen with the first click. Based on participants’ data, we calculated hypothetical costs of each option for each participant, summing the time needed for the first click and the time for the remaining clicks needed to reach each option.

**Figure 2:**
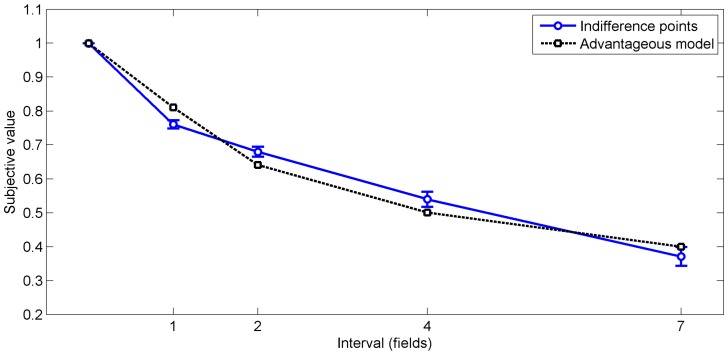
Temporal discounting. Indifference points mark the subjective value at each interval between soon/small and late/large option. The advantageous choice model shows the discounting for choosing always the option with the best time/money ratio. Error bars indicate standard errors of the mean over participants.

After determining the advantageous choices for each trial based on this cost/benefit ratio, we computed individual discounting functions based on the choices of the participants. For all trials of each interval, we fitted a logistic function to the choices (SS/LL) depending on the different reward ratios between the SS and the LL option [25]. The point of inflection of the logistic function determined the point of indifference for each interval (see Figure 2), where the two options were of equal subjective value [18]. We determined indifference points for modeled advantageous choices and for real choices of each participant (for details, see Material S1), yielding two discounting curves across intervals.

Participants’ indifference points showed significant discounting (ANOVA with the factor interval, *F*(3, 72) = 190.68, *P*<0,001, *η_p_^2^* = 0.89). Comparing modeled indifference points to real indifference points yielded a strong correlation (Mean *R^2^* = 0.93, *SD* = 0.09). Across participants, 76.53% (*SD* = 5.5%) of real choices were classified as advantageous according to the model. Of the sooner/smaller choices, 63.3% (*SD* = 8.6%) were advantageous, while of later/larger choices 83.97% (*SD* = 3.47%) were advantageous, yielding a significant expected bias of disadvantageous choices being sooner/smaller choices of 20.67% (*SD* = 9.6%, Wilcoxon signed rank test, *Z* = 4.37, *P*<0.001, *r* = 0.17).

To further quantify participants’ performance in terms of money lost, we compared the real choice scenario with counterfactual scenarios where participants could have chosen a) only advantageously, b) only SS, and c) only LL. Notably, these counterfactual scenarios considered the resulting changes in trial durations and were always calculated to match the overall given time of 3×8 minutes. Hence, rewards for each scenario were calculated per unit time and extrapolated to overall given time of 3×8 minutes. First, we calculated the hypothetical amount of money that could have been collected within the given overall time if participants had only chosen advantageously (for details, see Material S1). On average, participants gained 245 Cent (*SE = *5.7 Cent). Compared to the hypothetical gain of choosing only advantageous (*M* = 256 Cent, *SE = *5.5 Cent), participants lost 10 Cent (*SE = *1.54 Cent) that could have been collected additionally in the given amount of time if participants had chosen advantageously (*t*(24) = 7.06, *P*<0.01). Second, calculating the hypothetical gain for choosing only SS or LL options revealed that overall such simple strategies would have led to less gain. Within the given overall time, however, a pure LL strategy would have yielded a much higher gain (M = 246 Cent, SE = 27.8 Cent) than a pure SS strategy (M = 131 Cent, SE = 11.9 Cent). This further indicates that within the paradigm, participants’ bias to the SS option lead to disadvantageous decision making.

To validate our assumption that options’ values and the invested time were the main factors influencing participants’ decision, we aimed to exclude the possibility that choosing the LL choice simply included a higher effort in planning, which might be aversive, leading to a disadvantageous SS choice. We reasoned that due to its higher distance, the LL option had a higher probability of being placed diagonally to the avatar instead of being placed on a straight line. This could lead to a higher effort in planning the way to reach the LL option, since diagonal ways offer many different paths to be gone. To exclude this, we correlated the diagonality of each LL option (diagonality = min[DistanceX,DistanceY]/max[DistanceX,DistanceY] → diagonality = 0 for straight lines and diagonality = 1 for diagonally (45°) placed options) with the decision time of the first click, reasoning that increased decision difficulty due to planning effort should also increase decision time. However, the correlations showed no significant effects (*M*(*r* ^2^) = 0.05, *SE*(*r* ^2^) = 0.01, *M*(*p*) = 0.37, *SE*(*p*) = 0.06), indicating that planning effort did not play an influential role on choosing options.

**Figure 3:**
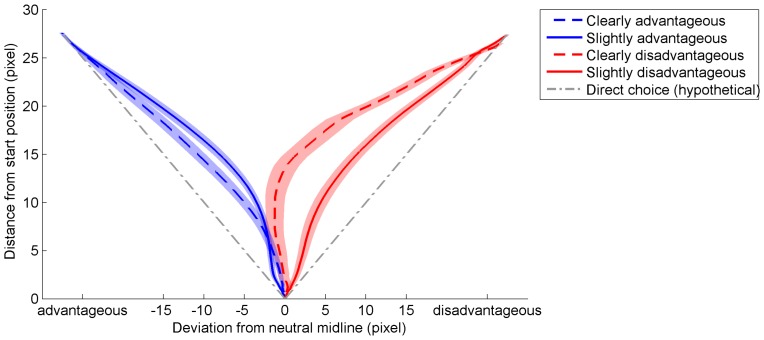
Mouse movement trajectories. Movements reach from the starting location at the begin of a trial to the first click into a movement field, leading to advantageous (here: left) or disadvantageous (here: right) choices. Direct choice paths mark the shortest way to the movement field. Deflection of trajectories from the direct choice path to the neutral midline between two movement fields indicates conflict in the decision process. Conflict is lowest for clearly advantageous choices and highest for clearly disadvantageous choices. Shaded areas mark standard errors.

We expected mouse movement trajectories to yield further information about participants’ choice behavior, especially for disadvantageous choices. Hence, we analyzed mouse movement trajectories [13], [15] to the first movement field (for details, see Material S1). Trajectories were normalized in length to 33 time-slices per trial (see Material S1), aligned to common starting position, and rotated to match a setup as used in previous mouse movement studies [15], [18], with a central starting position and targets representing different choices in a left and a right position. Advantageous choices were assigned to the left target; disadvantageous ones to the right (see Figure 3). Since mouse movements reflect the tendency of choice at each moment of time, deflections of the movement to the (finally) not chosen option indicate conflict through the decision process [15]. We categorized choices as advantageous and disadvantageous according to the model. Furthermore, we distinguished choices with clear utility difference and slight utility difference [26]. For the latter distinction, we performed two steps. First, we calculated the distance of each trial to the indifference point of each participant: For each trial of a specific delay, we calculated the difference between the respective indifference point and the small/large reward ratio of the trial. Second, we categorized all trials by performing a median split on this distance to the indifference point. Hence, trials were categorized as yielding a slight utility difference (low distance to the indifference point, *M* = 0.14, *SD* = 0.01) or as yielding a clear utility difference (high distance to the indifference point, *M* = 0.47, *SD* = 0.02).

Together, this yielded 4 types of choices, with different numbers of trials per type: slightly advantageous (*M* = 110.2 trials, *SE* = 4.16 trials), clearly advantageous (*M* = 148.72 trials, *SE* = 4.11 trials), slightly disadvantageous (*M* = 67.4 trials, *SE* = 2.57 trials), clearly disadvantageous (*M* = 11.52 trials, *SE* = 1.72 trials). To minimize the possibility of outliers influencing the averaged data especially for the conditions with fewer trials, we performed the following analysis on median movement deflection of each condition. An ANOVA with the factors utility difference (clear/slight) and choice (advantageous/disadvantageous) yielded no significant main effects (all *P*>0.35), but the expected interaction (*F*(1, 24) = 4.55, *P*<0.05, *η_p_^2^* = 0.16; see Figure 3): advantageous choices showed the largest deflection when utility differed only slightly; disadvantageous choices showed the overall largest deflection when utility differed clearly. Hence, when choosing disadvantageously, participants’ experienced higher conflict, especially if the utility difference between the options was clear.

**Figure 4:**
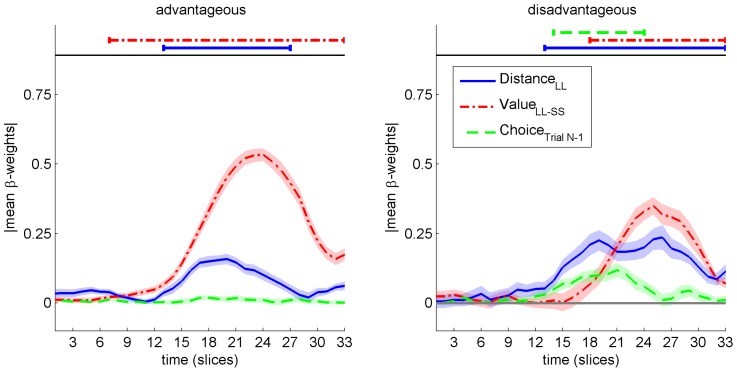
Beta-weights from time-continuous multiple regression analysis. Beta-weights represent the different influences on the mouse movement angle on the XY plane (shaded areas around the curves indicate the standard error of beta-weights for each time-slice). Left, beta-weights for advantageous choices. Right, beta-weights for disadvantageous choices. Above each graph, consecutive time-slices with a significant difference from zero (8 consecutive *t*-tests) are marked for each beta-weight.

To get further information about the sub-processes, leading to disadvantageous choices and increased conflict therein, we applied a time-continuous multiple regression to mouse trajectories, as we successfully used earlier for perceptual decisions [15] and intertemporal decisions [18]. To this means, we first calculated for each time-slice the movement angle relative to the y-axis. Movement angle has two advantages over raw X/Y-movements: First, it better reflects the instantaneous tendency of the mouse movement since it is based on a differential measure rather than the time-cumulative effects in raw trajectory data; second, it integrates the two measures of movements on the XY-plane into a single measure. Since we aimed to dissect different influences on mouse movements, we then applied a three step procedure to the movement angle, separately for the advantageous and the disadvantageous choices [15]. In the first step, we coded for each participant three predictors for all trials: *distance to the late option*, *value differences*, and *previous choice* (to avoid multicolinearity, we checked that variance inflation factors stayed below 1.5 for all regressors in all conditions and participants). To derive comparable beta-weights in the next step, we normalized the range of each predictor to a range of −1 to 1. In the second step, we computed multiple regressions with these predictors (33 time-slices, hence 33 multiple regressions) on the movement angle. This yielded 3 time-varying beta-weights (3 weights×33 time-slices) for each participant. Finally, in the third step, we computed grand averages of these 3 time-varying beta-weights yielding a time-varying strength of influence curve for each predictor in the 2 conditions (see Figure 4).

To quantify the strength of influence reflected by the beta-weights, we performed consecutive t-tests against 0, indicating which temporal segments showed significant influences., Notably, to compensate for multiple comparisons of temporally dependent data, we chose as criterion of reliability a minimum number of 8 consecutive significant *t*-tests (as indicated by Monte Carlo analyses on this issue [27]).

For advantageous choices (see figure 4, left), the beta-weights for the predictor *distance to late option* showed a significant influence between time-slices 13 to 27 (*M*(time) = 328 ms–680 ms); for the predictor *value differences*, beta-weights showed a significant influence between time-slices 7 to 33 (*M*(time) = 177 ms–832 ms); for the predictor *previous choice*, no significant influence was present.

For disadvantageous choices (see figure 4, right), the beta-weights for the predictor *distance to late option* showed a similar significant influence in the direction of the SS option (positive angle) between time-slices 13 to 33 (*M*(time) = 349 ms–887 ms); for the predictor *value differences*, beta-weights showed a shortened significant influence between time-slices 18 to 33 (*M*(time) = 483 ms–832 ms); interestingly, for the predictor *previous choice*, beta-weights now showed a significant influence of the previous choice between time-slices 14 to 24 (*M*(time) = 376 ms–645 ms).

To quantify the differences between the two conditions, we calculated the area under the curve for each beta-weight, yielding one value for each predictor and condition. To test the differences for statistical significance, we used a *t*-test based jackknifing procedure as has been used previously for mouse movement data [15] and lateralized readiness potentials (LRP) (see [28] and Material S1). The *distance to late option* showed significant differences, with advantageous (*M = *1.51, *STE* = 0.2) choices indicating less weighing of this dimension than disadvantageous (*M = *3.52, *STE* = 0.4) choices (*t*(24) = 4.85, *P*<0.001). For *value differences*, this reversed significantly, with advantageous (*M = *7.12, *STE* = 0.2) choices indicating more weighing of this dimension than disadvantageous (*M = *3.52, *STE* = 0.43) choices (*t*(24) = 8.26, *P*<0.001). For *previous choice*, a significant increase from advantageous (*M = *0.16, *STE* = 0.11) choices to disadvantageous (*M = *1.21, *STE* = 0.3) choices (*t*(24) = 3.75, *P*<0.01) indicates that this dimension was only influential in the latter choices. From this time-continuous multiple regression analysis, we conclude that participants undervalued the value information in disadvantageous trials, in favour of the distance information and, additionally, were biased by their previous choice to a choice-repetition.

To finally validate this choice-repetition bias, we performed a sequential analysis across choices, counting the occurrence of SS-SS, SS-LL, LL-LL, and LL-SS pairs of trials, separately for advantageous and disadvantageous trials. For disadvantageous trials, we found the expected choice repetition bias, with more frequent SS-SS (52.96%) pairs than LL-SS (42.43%) pairs, and more frequent LL-LL (57.57%) pairs than SS-LL (47.04%) pairs. This bias vanished for advantageous trials, with almost equally frequent SS-SS (26.96%) pairs and LL-SS (24.97%) pairs, and similarly, almost equally frequent LL-LL (75.03%) pairs and SS-LL (73.04%) pairs. This observation was supported by a repeated measures analysis of variance (ANOVA) with the factors *choice_trial N_*, *choice_trialN+1_*, and *advantageousness*, indicating a significant three way interaction of these factors, *F*(1,24) = 21.83, *P*<0.001. To check, if the repetition bias developed across the time of the experiment, we performed the same ANOVA adding the factor block. While we found again the expected three way interaction, *F*(1,24) = 11.61, *P*<0.01, the only significant interaction with the factor *block* was a two way interaction with the number of SS/LL choices, *F*(2,48) = 4.402, *P*<0.05, indicating a slight decrease in SS choices across the experiment. All other interactions with the factor *block* were not significant (all *P*>0.12). Taken together, this supports our hypothesis that despite complete randomization of the options sequence and the placement of options, there was a significant constant repetition bias, but only for disadvantageous choices.

## Discussion

This study suggests a participant-specific and process-oriented approach to human decision behavior in intertemporal choice tasks. Using a novel paradigm, we compared modeled advantageous decisions and real participants’ decisions. While the largest part of decisions was advantageous, the remaining disadvantageous choices showed a bias towards the sooner/smaller option in concordance with previous research [20]. Tracing decision behavior [13], [15], [16] continuously, we analyzed properties of the decision process. Comparing the deflections of movements leading to choices, we found moderate conflict for advantageous and disadvantageous choices alike, when utilities differed only slightly, as could be expected due to increased task difficulty [21]. In contrast, we found high conflict for disadvantageous choices compared to advantageous choices when the utilities of the options differed clearly and, hence, choices should have been easy and clear-cut. We interpreted this finding as indicating that individuals chose disadvantageously against better knowledge, but further in depth analysis of the time course of movements revealed a more precise picture of the processes leading to these choices. While participants’ movements were influenced by both, the value information and the delay information simultaneously, in disadvantageous choices, delays became more influential, values became less influential, and the previous choice became influential, indicating a choice-repetition bias, constituting a possible source for the increased conflict. An additional sequential analysis indicated that this choice repetition bias for disadvantageous trials was constant across the experiment.

The present study advances research of intertemporal choice in three ways. First, within this study, we modeled advantageous choices according to individual behavior. This offers a classification of advantageous and disadvantageous choices without strong prior assumptions. Therefore, the comparison of discounting parameters estimated on the basis of choices with a rather hypothetical model of utility [2] is enriched by comparing advantageous and disadvantageous choices directly. Previous research revealed conflicting evidence concerning the discrimination of an impulsive system preferring immediate reward and a reflective system able to delay gratification (see e.g. [29], [11], but see also [25], [30]). The inconsistency of these results might be attributed to the fact, that sooner/smaller choices can be advantageous as well and then preferable to the reflective system. Hence, comparing advantageous and disadvantageous as well as sooner/smaller and later/larger choices, which was not feasible in the current study due to the resulting low number of trials in the disadvantageous condition, could further progress these investigations. Notably, our individual model of advantageous choices was based on the value/time ratio. Hence, it could be argued that later larger choices also included increased effort, which might have lead to even further discounting. While, this account cannot be excluded completely, it is, in fact, not contradicting our model of advantageous choices, since the given aim of participants was to optimize the amount of money within the given time. Therefore, it would be illuminating to vary effort related variables in the paradigm to extract possible detrimental influences of effort related discounting [31] on advantageous decision making in our paradigm.

Second, in the experiment, we observed decision processes continuously, opening a window on the weighing process to the final choice [15], [18]. These continuous data can help to substantiate models of intertemporal choice by understanding the mechanisms behind choice patterns [32]. Exemplarily, the increased deflection of choice trajectories observed through the decision process when utilities of the options differ clearly suggests that disadvantageous choices compared to advantageous choices are preceded by more conflict between the two possible responses (on erroneous responses and conflict, see [21]). While this supports the general assumption that disadvantageous options were chosen against better knowledge [10], [12], further in depth analysis provided more detailed information about the weighing of options’ properties in the decision process. First, value and time information were influential on choice behavior through similar time windows, providing evidence for a simultaneous evaluation of these two dimensions. Second, when choosing disadvantageously, weighing of the value information was less pronounced, in favor of the delay information, compared to choosing advantageously. Surprisingly, disadvantageous choices also showed a bias by the previous choice in the middle of the decision process. The latter finding seems surprising, since the randomized variation of the relative locations of both options exclude a simple response-repetition account. Instead, this effect that was constant across the whole experiment indicates that previous choices might lead to a bias of “choosing the same thing again”. Though speculative, similar behavior has been found previously for sequences of choices due to the cognitive system being caught in the attractor basis of a previous choice state [33] (see also [14]) for a theoretical discussion of this phenomenon). While such an interpretation certainly calls for further investigation, this pattern of influences indicates that when the weighing process of time and value was influenced by the previous decision, participants experienced stronger conflict and, hence, had a harder time in choosing than could be expected by the properties of the offered options.

Third, the presented computer game increases ecological validity (compare e.g. [34]) in three ways: First, through direct feedback of the achieved reward after each choice; second, through the real experience of short and long time delays instead of the presentation of abstract time delays; third, through an immersive scenario, engaging participants into a virtual world instead of an abstract scenario.

Besides these advances, the general comparability of our experimental approach with previous studies of intertemporal choice needs to be discussed. Previous studies offered options in the far future and time delays were presented in an abstract way. In our paradigm, options could be collected in real-time and time delays were represented by spatial distance. Despite these differences, our results indicate comparability in that we found discounting behaviour suggesting that participants weighed time and reward attributes for each option. As expected, participants also showed a bias towards sooner/smaller choices. Furthermore, within the wide range of intertemporal choice paradigms, using primary rewards [29], one-out-of-many-choices-rewards [11], [25], or hypothetical rewards [5], [8], [26], our approach is reasonable in so far as it implements real reward and a real experience of passing time. Finally, since our approach focuses on the differences between advantageous and disadvantageous choices, it is not only related to intertemporal choice, but integrates in the broad range of different multi-attribute choice paradigms, including also for example probability [35], effort [31] or dynamical discounting [36].

Taken together, our findings emphasize the value classifying advantageous decisions individually and tracing decision processes continuously to approach intertemporal choice and human choice behavior in general.

## Supporting Information

Material S1 **Additional methodological details.**
(PDF)
