# Supplementary material for: Harder than Expected: Increased Conflict in Clearly Disadvantageous Delayed Choices in a Computer Game
Source: PLoS One. 2013 Nov 15;8(11):e79310. doi: 10.1371/journal.pone.0079310 (PMC3829829; doi:10.1371/journal.pone.0079310)
Supplement: Material S1 — Additional methodological details. (PDF) [file pone.0079310.s001.pdf]

Harder than expected: increased conflict in clearly disadvantageous delayed choices in a computer game.

Supplementary Material S1

Stefan Scherbaum[1], Maja Dshemuchadse[1], Susanne Leiberg[2], Thomas Goschke[1]

[1]Technische Universität Dresden, [2]University of Zurich

Correspondence should be addressed to:

Stefan Scherbaum

Department of Psychology, Technische Universität Dresden

Zellescher Weg 17, 01062 Dresden, Germany

Phone: ++49 351 463 33598

E-mail: stefan.scherbaum@psychologie.tu-dresden.de

Additional methodological details

Procedure: Constraints for placing the two options

1. The distance of each option field to the avatar was defined by the design. The distance was defined as the number of fields the avatar had to be moved to reach the option.
2. The first move should always decrease the distance to one option but increase the distance to the other option so that it represents a preliminary decision for one option against the other.
3. The angle between the direct lines connecting the avatar with the options was constrained to be above 90° (see second constraint), but also below 135° to allow participants to start moving the mouse on a line between the two options and hence to decide while already moving the mouse.

Advantageous Choice Model

For each trial of each participant, we determined the advantageous choice by comparing cost/benefit ratios of both choice options. As cost, we took the time that was needed to reach the option. Hence, we summed up the time for the first click and the residual clicks until the participant reached the field containing the option. If the late option was chosen by the participant, we calculated the hypothetical cost for the early option by summing up only the clicks until the field with the early option would have been reached. If the early option was chosen by the participant, we calculated the hypothetical cost for the late option adding additional clicks equal to the duration of the last click to the sum of clicks until the field with the late option would have been reached. Taken together, we determined the advantageous choice by comparing the ratios

$$\frac{reward_{soon}}{time_{soon}} \text{ and } \frac{reward_{late}}{time_{late}} .$$

To estimate the hypothetical loss, we compared for each participant the real sum of collected rewards with the hypothetical win of choosing only advantageously. Hence, we first calculated the win per time unit for completely advantageous choices by dividing $win_{advantageous}$, the sum of possibly collected rewards if chosen advantageously for each trial, by $time_{advantageous}$, the amount of the respective time this would have taken. We then calculated the possible amount of reward, $win_{hypothetical}$ by multiplying this hypothetical win per time unit with the time participants had in reality, $time_{real}$:

$$win_{hypothetical} = \frac{win_{advantageous}}{time_{advantageous}} \cdot time_{real}.$$

Discounting & indifference points

As an estimate of the indifference point, we determined the point of inflection of a logistic function fitted to the individual choices (sooner/smaller vs. later/larger) as a function of increasing value differences (compare e.g. [1]). The fitting of the logistic regression model was performed using the StixBox mathematical toolbox by Anders Holtsberg (http://www.maths.lth.se/matstat/stixbox/). The fit was based on the model $log(p/(1-p)) = X*b$, where p is the probability that the choice is 1(sooner option) and not 0 (later option), X representing value differences, and b representing the point estimates for the logistic function.

Mouse movement trajectories

Mouse movement trajectories were processed in four steps.

1. Each trials trajectory was aligned for common starting position: $x(t) = x(t) – x(1); y(t) = y(t) – y(1)$.
2. Each trajectory was rotated so that the starting (the middle of the avatar's starting field) and the end point (the position of the first click) of the trajectory were positioned in a way

similar to previous mouse studies (compare e.g. [2,3]) with the start field in the center of the bottom of the screen and the target on the upper left side of the screen.

3. Where necessary, trajectories were mirrored so that advantageous choice movements always aimed to the upper left and disadvantageous movements always aimed to the upper right target. Fourth, each trajectory was time-normalized to 33 equal time slices to make the trajectories of different length comparable and analyzable [4,5]. We chose 33 time-slices to approximately match the mean number of samples for mouse movements, defined by the mean decision movement time of 770 ms and the mouse sampling rate of 50 Hz. Noteably, results did not change when varying the number of time-slices within a reasonable rangte (e.g. 50 time-slices).
4. Only trials in which the first decision (represented by the first click) and the final decision (represented by the collected reward) matched were included in the analysis (99%, *SD* = 0.7%). For statistical analysis, we determined the deflection of each movement by calculating the area under the curve between the real movement and a straight line from the start to the target position [4].

To validate the hypothesis that mouse trajectories did reflect the complete process of decision making and to exclude that decisive parts of the decision making process took place before participants moved the mouse, we also analyzed movement initiation time, defined as the time between stimulus onset and the time when the position of the mouse diverging more than 2 pixels from the starting position. Performing the same ANOVA as we did for movement deflection on movement initiation time (*M* = 262.57 ms, *SE* = 23.98 ms), there was no significant effect.

Jackknife based analysis of mouse movement trajectories

Jackknifing represents one of several possible resampling methods: for each dataset d in a group of n datasets, the jackknife procedure produces a new mean dataset consisting of all datasets in the group, except dataset d. Hence, for dataset 1, the method creates a mean dataset averaging across the data in datasets (2, 3, …, n). For dataset 2, it creates a mean dataset averaging across the data in datasets (1, 3,4,…,n). While this reduces the noise occurring in time-series data, e.g. LRP data, it also reduces the degrees of freedom. Hence, for statistical testing, test parameters have to be adjusted (for further details,see [6]).

References

1. Ballard K, Knutson B (2009) Dissociable neural representations of future reward magnitude and delay during temporal discounting. Neuroimage 45: 143–150.

2. Scherbaum S, Dshemuchadse M, Fischer R, Goschke T (2010) How decisions evolve: The temporal dynamics of action selection. Cognition 115: 407–416.

3. Dshemuchadse M, Scherbaum S, Goschke T (2012) How Decisions Emerge: Action Dynamics in Intertemporal Decision Making. Journal of experimental psychology General.

4. Spivey MJ, Grosjean M, Knoblich G (2005) Continuous attraction toward phonological competitors. Proceedings of the National Academy of Sciences 102: 10393–10398.

5. Scherbaum S, Dshemuchadse M, Fischer R, Goschke T (2010) How decisions evolve: The temporal dynamics of action selection. Cognition 115: 407–416.

6. Miller J, Patterson T, Ulrich R (2001) Jackknife-based method for measuring LRP onset latency differences. Psychophysiology 35: 99–115.
